# Before Mr. Justice Ridley and a Special Jury

**Published:** 1916-03-18

**Authors:** 


### MACMILLAN AND CO. (LIMITED) AND ANOTHER v. THE NURSING PRESS (LIMITED) AND OTHERS.

### (Before Mr. Justice Ridley and a Special Jury.)

In this case Messrs. Macmillan and Co., Ltd., and Miss Swanhilde Bulan claimed damages for libel against the Nursing Press, Ltd., Mrs. Ethel Gordon Fenwick, and Press Printers, Ltd.

The defendants admitted the publication, but said that the words were true and were fair comment.

The plaintiffs alleged express malice.

Mr. Dickens, K.C., and Mr. McCardie appeared for the plaintiffs; and Mr. Gordon Hewart, K.C., and the Hon. M. M. Macnaghten for the defendants.

Mr. Dickens said that Macmillan and Co., the publishers, were the proprietors of the *Nursing Times*, of which Miss Bulan was the editor. The defendant Mrs. Ethel Gordon Fenwick, with her husband, owned nearly all the shares in the Nursing Press, Ltd., who owned the *British Journal of Nursing*. The libel appeared in the defendants' newspaper of May 15, 1915. It assumed the veil of patriotism, but its real object was to crush the *Nursing Times*. Miss Bulan was chosen as a victim because her father was a German, who, however, had left Germany in 1875. The libel was as follows:—

"Fräulein Bulau,—We beg to inform British nurses at home and abroad that, in spite of the determined silence of Macmillan and Co., we have now in our possession documentary evidence that their publication, the *Nursing Times*, is edited by Fräulein Bulau, the untrained lady of German parentage who, under an assumed name, has been resident in England for some years, who was hurriedly naturalised as a British subject three months after the declaration of war, and who has done us the honour of attempting to control our professional affairs in England—of course for adequate financial consideration.

"This significant fact having been established, we may now proceed to invite replies to the following questions from all and sundry whom they may concern:—

"To the Secretary of State for War,—Is the Matron-in-Chief of the Territorial Force Nursing Service still collaborating with Fräulein Bulau in the production of the *Nursing Times*, and, as heretofore, is she attending at its office in a subordinate position and therefore under the control of its German editor? . . .

"To Queen's Nurses,—When your Penny Pension Scheme was started for advertising purposes from the office of the *Nursing Times* did you know that the hon. secretary of the fund was Fräulein Bulau, and not Miss Bulan? . . .

"To British Nurses all the World Over,—It is to be presumed that your very nearest and dearest are fighting for King, country, liberty, and honour a most desperately, callous, and cruel foe. . . . Not one penny of clean British money should subsidise the publication in question . . . until the proprietors have eliminated from its management German exploitation at your expense."

He (counsel) submitted that the only meaning of the word "assumed name" and "hurriedly naturalised" was that Miss Bulan was a spy.

The Penny Pension Scheme, which had realised about £1,100, was started by the nurses themselves, and Sir Frederick Macmillan was asked to take it up. Miss Bulan was born in Alsace in 1874. In 1875 her father came to England, and on his return expressed the opinion that England was the only place where one could be really free. Miss Bulan had only been to Germany three times since 1876, and she was there treated as a British subject. She became a journalist; she had been sub-editor of the *Churchwoman*, and had worked for *Our Hospitals and Charities*. The *British Journal of Nursing* was very aggressive on the subject of the registration of nurses, and so it came into conflict with many nursing authorities. The *Nursing Times* refused to take sides in any political questions, and was therefore attacked. The matron of the Territorial Force Nursing Service, Miss Sidney Browne, simply helped in dealing with nursing subjects in which she was an expert.

### Miss Bulan's Evidence.

Miss Bulan, examined by Mr. McCardie, bore out counsel's statement.

In cross-examination by Mr. Macnaghten, she said that she was of pure German descent on both sides. Her naturalisation certificate called her "an alien" in the name "Bulau." She had never taken any steps to denationalise herself in Germany, but by German law absence for ten years denationalised her automatically.

She had had no training as a nurse, nor had she ever professed to have. The question of the registration of nurses had become very important, though the great bulk of nurses were indifferent.

Mr. Macnaghten, in addressing the jury, said that Mrs. Fenwick had been a trained nurse. She had taken a prominent part in the movement for registration, and from first to last had not made one penny out of the paper. She felt that responsible people could not be neutral, and that the plaintiff's paper in taking no side must be deemed to be hostile. She thought also that the editor of a nursing paper ought to have nursing qualifications.

Mrs. Fenwick said that she was the wife of Dr. Bedford Fenwick, of 20 Upper Wimpole Street. She had devoted her life to the betterment of nurses. She had formed the opinion that they were overworked and underpaid, and she tried to get them a legal status, without which paid workers were in a very dependent position. She and her husband held all the ordinary shares (7,000) in her paper, but they had never received dividends. She had always thought that Miss Bulan was a Swede. When she saw the certificate she thought that she ought to let the nursing profession know.

In cross-examination by Mr. Dickens, she said that her paper appealed to the educated nurses; the plaintiff's to the uneducated—the wardmaids. She did not suggest that Miss Bulan was a spy. On April 17, 1915, she had written in her paper praising a lady, chairman of a nursing committee on which she sat. That lady was of German birth. They had made her resign.

Mr. Dickens: Did she resign after this action was begun?—Yes.

Continuing, the witness said that she had written of "dear Sister Agnes Karll and our many German sisters" because they were doing their duty. Sister Karll had nothing to do with the *Nursing Times*. She did not think that she had attacked Miss Bulan; it was only criticism. She thought that the Penny Pension Scheme was promoted by the plaintiffs as an advertising scheme.

In re-examination, the witness said that she and her husband had sunk £10,000 in her paper.

Counsel addressed the jury, and his Lordship summed up.

The jury returned a verdict for the plaintiffs, with £500 damages. The defendants gave an undertaking not to publish in future anything similar to the words complained of. [*From the "Times" report.*]

*For Judge's Summing-up see next page.*

